# Gut microbiota dynamics and its impact on the efficacy of ACTH therapy in infantile epileptic spasms syndrome

**DOI:** 10.3389/fneur.2026.1804171

**Published:** 2026-04-15

**Authors:** Xuan Zhang, Xinyue Hu, Xiaosheng Hao, Hongbo Zhang, Zhiming Tao, Jianmin Liang

**Affiliations:** 1 Department of Pediatric Neurology, Children’s Medical Center, The First Hospital of Jilin University, Changchun, China; 2 Jilin Provincial Key Laboratory of Pediatric Neurology, Changchun, China; 3 Neuromedical Center, First Hospital of Jilin University, Changchun, China

**Keywords:** 16S rDNA, brain-intestine axis, focal epilepsy, gut microbiota, infantile epileptic spasm syndrome

## Abstract

**Background:**

Infantile epileptic spasms syndrome (IESS) is a severe early-life epileptic encephalopathy with diverse etiologies, yet its precise pathogenesis remains unclear. The gut–brain axis has emerged as a key modulator of neurodevelopment and seizure susceptibility, and although gut microbiota dysbiosis has been implicated in various epilepsy syndromes, its role in IESS, particularly in relation to treatment response, remains unexplored. This preliminary study therefore investigated gut microbiota composition in pediatric IESS and its potential as a biomarker of response to adrenocorticotropic hormone (ACTH) therapy.

**Methods:**

Eighteen patients with IESS were enrolled. Two age-matched control groups were established: a focal epilepsy (FE) group and a healthy (H) group. The 18 patients with IESS were subsequently categorized into two groups based on therapeutic response: ACTH-effective and ACTH-ineffective. Fecal samples were collected before and after ACTH treatment and subjected to 16S ribosomal DNA sequencing, followed by bioinformatics analyses.

**Results:**

Gut microbiota α-diversity was significantly lower in the IESS group than in the FE group, and β-diversity significantly differed among the IESS, FE, and H groups. Proteobacteria and Actinobacteria exhibited significantly higher relative abundances in the IESS group, while Ruminococcaceae was significantly lower than in the FE and H groups. The IESS group had significantly higher Veillonellaceae abundance but significantly lower Lachnospiraceae and Faecalibacterium abundances compared with the H group. Intestinal microbiota network structure was weaker in IESS than in the FE and H groups. Before ACTH treatment, the ACTH-ineffective subgroup exhibited significantly lower actinomycetes and bifidobacteria abundances, downregulated amino acid metabolism pathways, and weaker network aggregation than the effective subgroup. After treatment, the IESS group displayed enhanced network aggregation, significantly decreased Bacillus abundance, and downregulated retinol metabolism. Additionally, Corynebacteriales and Enterobacteriaceae were enriched before treatment, while Intestinibacter was enriched after treatment.

**Conclusion:**

Gut microbiota profiles in children with IESS suggested a potential association with response to ACTH therapy. These preliminary findings, while requiring validation in larger independent cohorts, highlight a potential role for the gut microbiota in the pathophysiology of IESS and underscore the need for further multi-omic investigations to elucidate underlying mechanisms.

## 1 Introduction

Infantile epileptic spasms syndrome (IESS) is a severe early-life epileptic encephalopathy characterized by clusters of nodding- and hugging-like spasms. The incidence rate is approximately 0.31 per 1,000 live births, with marked clinical heterogeneity and therapeutic challenges (1). More than 200 possible causes of IESS have been identified, and the condition may develop during the prenatal, perinatal, or postnatal stages (2). Although over 64% of cases can be attributed to identifiable causes (acquired structural abnormalities in 22.4% of cases (2), genetic factors in 14.4% (3), and congenital structural abnormalities in 10.8% (3)), the specific pathogenesis remains incompletely elucidated (4). Multiple hypotheses have been offered for its pathogenesis, including prenatal stress exposure (Zou’s hypothesis), hypothalamic–pituitary–adrenal (HPA) axis dysfunction, asynchronous development theory, N-methyl-D-aspartate receptor hypothesis, 5-hydroxytryptamine hypothesis, cortical–subcortical interaction hypothesis, brainstem dysfunction, immune dysfunction, and gene variation (5, 6).

In recent years, the role of the gut–brain axis in epilepsy has garnered significant attention. The gut microbiota interacts with the central nervous system through multiple pathways, including neural, endocrine, and immune mechanisms. These interactions involve at least six possible regulatory pathways between the gut microbiota and epilepsy: gut–brain neural network regulation; HPA axis modulation; regulation of the intestinal immune system; intestinal microbiota synthesis of neurotransmitters and neuromodulators; the endogenous cannabinoid mechanism; and regulation of the intestinal mucosal barrier and blood–brain barrier (7). This establishes a fundamental theoretical framework for understanding the microbiota–gut–brain axis. Notably, abnormal gut microbiota may also have an impact on HPA axis hormone or cytokine levels (8, 9), and gut microbiota characteristics in patients with IESS differ significantly from those in healthy individuals (10). Moreover, the antiepileptic effects of a ketogenic diet may also involve mechanisms modulating the gut microbiota (11).

Emerging evidence suggests that immune dysregulation may play a role in the pathogenesis of IESS. Although rare, spontaneous seizure remission following viral infections, including human herpesvirus 6, human herpesvirus 7, and certain respiratory viruses, has been reported (12–18). The proposed mechanism involves infection-induced immune modulation: while proinflammatory cytokines (e.g., IL-6, IL-17A, IL-1β) are associated with epilepsy onset and progression (19, 20), immunosuppressive cytokines (e.g., IL-10, TGF-β) may promote symptom remission (21). Notably, the gut microbiota is a critical regulator of host immune homeostasis, and its composition has been shown to influence cytokine profiles and immune responses (8, 9). Given this shared capacity for immune modulation, it is plausible that the gut microbiota may similarly contribute to the immunopathological processes underlying IESS, potentially through pathways overlapping with those involved in virus-induced remission.

Collectively, these observations suggest that the gut microbiota may play a key role in IESS pathophysiology, potentially influencing disease course and treatment response through immunomodulatory mechanisms. However, research exploring the association between the gut microbiota and IESS remains limited. In this preliminary study, we aimed to characterize gut microbiota dynamics in children with IESS using 16S rDNA sequencing and to explore their potential as biomarkers of response to ACTH therapy, with the goal of providing mechanistic insights into IESS and informing treatment optimization.

## 2 Methods

### 2.1 Subjects and sample collection

This study was approved by the Research Ethics Committee of the First Bethune Hospital of Jilin University (Approval Number: 24K097-001). A total of 54 children aged 0 to 24 months were enrolled between December 2023 and December 2024, comprising 18 children with IESS (IESS group), 18 children with focal epilepsy (FE group), and 18 healthy infants (H group). Children with focal epilepsy were included as a disease control group, as focal seizures represent the most common seizure type in the pediatric population.

Children in the IESS group received natural adrenocorticotropic hormone (ACTH) via intravenous infusion at a dosage of 25 U once daily for 4 consecutive weeks. The 4-week regimen was adopted based on clinical considerations, including incomplete response to initial therapy or the presence of severe underlying etiologies. No tapering scheme was applied; ACTH was discontinued directly upon completion of the 4-week course. All patients were hospitalized during the treatment period for safety monitoring, including blood pressure, serum electrolytes, blood glucose, and signs of infection. Fecal specimens were obtained at two time points: within 24 h prior to the initiation of ACTH therapy (baseline, pre-treatment) and within 24 h after the completion of the 4-week ACTH course (post-treatment). A 0.2 g mid-stool sample was collected from each child using a sterile cotton swab and a sterile test tube, placed in an appropriately numbered pouch, stored at −80 °C, and transported on dry ice to a testing facility for analysis.

Based on treatment response, children with IESS were divided into two subgroups: the ACTH-effective subgroup, defined as no seizures with improvement in electroencephalography or a reduction in seizure frequency of at least 50%, and the ACTH-ineffective subgroup, defined as a reduction in seizure frequency of less than 50% or no improvement in electroencephalography. Patients were excluded if they had received antibiotics or probiotics within 2 weeks prior to enrollment or had a history of any other disease. All patients with epilepsy were diagnosed for the first time and had not been treated with antiepileptic drugs before enrollment. Children with IESS were included regardless of etiology; most had unknown etiology, while a minority presented with structural abnormalities on MRI or identified genetic causes. Additionally, clinical data including sex, age, gestational age, feeding method, seizure characteristics, and electroencephalography results were collected for all participants.

### 2.2 Gut microbiota 16S rDNA sequencing

16S rDNA amplicon sequencing was performed by Jilin Hehe Medical Testing Co., Ltd. A high-throughput sequencing library of the V3–V4 region of the 16S rDNA gene was constructed using a two-step amplification method using the amplification primers: 341F (5′-CCTACGGGGNBGCASCAG-3′) and 805R (5′-GACTACNVGGGTATCTAATCC-3′). The first-step PCR reaction system comprised 12.5 μL of KAPAHiFi 2X Mix, 0.5 μL of forward or reverse primer (10 μM), and 50 ng of template DNA in a final volume of 25 μL. The PCR reaction conditions were as follows: 55 °C for 30 s, 72 °C for 30 s, 25 cycles; and 72 °C for 5 min. 0.8 × AFTMag NGS DNA Clean Beads were used to purify the PCR products. The second amplification step was used to add different index sequences to each sample and to add all the sequence information needed for complete Illumina sequencing. The PCR reaction system comprised 10 μL KAPAHiFi 2X Mix, 1.5 μL of forward and reverse primers (5 pM), 5 μL of the purified first amplification product, and sterile water in a total volume of 20 μL. The reaction conditions were 95 °C for 3 min; 95 °C for 30 s, 55 °C for 30 s, 72 °C for 30 s, 8 cycles; and 72 °C for 5 min. Subsequently, the amplification products were purified using 0.8 × AFTMag NGS DNA Clean Beads for library construction.

QC-qualified 16S rDNA gene libraries were sequenced using the Pair-end 250 mode of the Illumina NovaSeq 6000 sequencing platform, and the resulting data were filtered for subsequent bioinformatics analysis.

Fast length adjustment of short reads was used to splice the bipartite sequences obtained from sequencing into one target region sequence. The target sequence was subjected to quality control filtering (fastq_quality_filter (-p 90 -q 25 -Q33) in FASTX-Toolkit 0.0.14), and the filtered sequence was compared with the reference database USEARCH 64 bit v8.0.1517 to remove chimeric sequences and obtain the final optimized sequence. The number of reads per sample was normalized by random subsampling to the minimum read count across all samples. Based on a 97% sequence similarity level, operational taxonomic unit (OTU) clustering analysis was performed using the Uclust algorithm in the QIIME software package. Subsequently, OTUs were annotated for species taxonomy based on the SILVA reference database.

### 2.3 Bioinformatics analysis of gut microbiota

Bioinformatics methods were used to cluster the effective sequencing data into operational taxonomic units (OTUs) and to optimize data processing. The relative abundance of each taxonomic unit was calculated at six phylogenetic levels (phylum, class, order, family, genus, and species), and the compositional spectrum of the microbial community was mapped. Chao1, ACE, observed species, Shannon, Simpson, and Good’s coverage indices were applied to assess the α-diversity of the gut microbiota. The Bray–Curtis dissimilarity coefficient and the weighted/unweighted UniFrac algorithm were used to construct a β-diversity matrix between samples by calculating community structure dissimilarity. Based on the linear discriminant analysis effect size (LEfSe) algorithm, an intergroup difference analysis model was constructed, integrating the non-parametric Kruskal–Wallis test and linear discriminant analysis (LDA) to screen for significant microbial taxa. Metastats is a method for comparing the variability of species contained in two sets of samples. The Phylogenetic Investigation of Communities by Reconstruction of Unobserved States 2 (PICRUSt2) functional prediction system was used to construct a gene function prediction model for the entire microbial community, based on the evolutionary correlation of phylogenetic marker genes. Sequencing data were matched with a reference database for functional annotation to predict the metabolic pathways and molecular functions of the microbial communities. Based on a non-parametric statistical method, we calculated the species abundance correlations, screened statistically significant Spearman association pairs, and constructed a microbial co-occurrence network topology model. Visualization was used to reveal the synergistic/antagonistic modes of microbial action and provide a theoretical basis for the analysis of community assembly mechanisms and disease-related bacterial interactions.

### 2.4 Statistical analysis

Clinical data of children in the IESS group and two control groups (FE group and H group) were collated using Excel and analyzed and charted using the following statistical software packages: SPSS 26.0 (SPSS, Chicago, IL, United States), “mixOmics” and “vegan” in the R environment, PICRUSt software, STAMP software, and the Micromedicine Biochemistry Cloud online platform. Fisher’s exact test was used to compare data between groups; the Shapiro–Wilk test was used to analyze the normality of the data (significance level of *α* = 0.05); the independent sample *t*-test was used to analyze normally distributed data; and the Mann–Whitney *U* test was used to analyze non-normally distributed data. In the comparison of data before and after treatment, paired *t*-tests were used for normally distributed data, and paired rank sum test (Wilcoxon signed rank test) was used for non-normally distributed data. Effect sizes were calculated using Hedges’ *g* for parametric comparisons and Cliff’s delta for non-parametric comparisons, with 95% confidence intervals (CIs) estimated via the non-central *t*-distribution or bootstrap methods, respectively. Correlation analysis was performed using Spearman’s rank correlation analysis. All statistical analyses were performed with *p* < 0.05 as the criterion for statistically significant differences.

## 3 Results

### 3.1 Characteristics of the study population

As shown in Table 1, there were no significant differences among the three groups in demographic characteristics (*p* > 0.05 for all). No significant differences were found in MRI structural abnormalities or genetic etiologies between the IESS and FE groups (*p* > 0.05 for each) (Table 1). Detailed seizure characteristics and EEG findings, including age at seizure onset, semiology, duration, frequency, and EEG localization, are provided in Supplementary Table 1.

**Table 1:** General characteristics of the infantile epileptic spasms syndrome (IESS), focal epilepsy (FE) control, and healthy control (H) groups.

Variables	IESS (*n* = 18)	FE (*n* = 18)	H (*n* = 18)	*p*-value
Demographics				
Birth weight (g), mean ± SD	2.99 ± 0.73	3.10 ± 0.24	3.38 ± 0.53	0.090
Age (months), mean ± SD	10.72 ± 5.07	13.72 ± 6.27	12.00 ± 7.42	0.360
Gender, *n* (%)				
Males	11 (61.1)	8 (44.4)	8 (44.4)	0.431
Females	7 (38.9)	10 (55.6)	10 (55.6)	0.431
Gestational age, *n* (%)				
Premature labor	4 (22.2)	3 (16.7)	1 (5.6)	0.334
Full-term gestation	14 (77.8)	15 (83.3)	17 (94.4)	0.334
Mode of delivery, *n* (%)				
Natural delivery	3 (16.7)	8 (44.4)	5 (27.8)	0.190
Cesarean section	15 (83.3)	10 (55.6)	13 (72.2)	0.190
Feeding situation, *n* (%)				
Breastfeeding	6 (33.3)	6 (33.3)	7 (38.9)	0.919
Artificial feeding	7 (38.9)	8 (44.4)	4 (22.2)	0.385
Mixed	5 (27.8)	4 (22.2)	7 (38.9)	0.569
History of intrauterine distress asphyxia, *n* (%)				
Yes	4 (22.2)	1 (5.6)	1 (5.6)	0.158
No	14 (77.8)	17 (94.4)	17 (94.4)	0.158
Etiology				
Structural abnormality on MRI, *n* (%)	4 (22.2)	2 (11.1)	—	0.657
Genetic etiology, *n* (%)	2 (11.1)	2 (11.1)	—	1.000

Data are presented as mean ± SD or *n* (%). IESS, infantile epileptic spasms syndrome; FE, focal epilepsy; H, healthy control; MRI, magnetic resonance imaging. *p*-values were calculated using the Kruskal–Wallis test for continuous variables and Fisher’s exact test for categorical variables. Detailed seizure characteristics and EEG findings are presented in Supplementary Table 1.

In the IESS group, fecal samples were not collected for three patients after ACTH treatment; thus, 15 cases were included in the comparison of gut microbiota before and after treatment. The IESS group was divided into ACTH-effective (*n* = 13) and ACTH-ineffective (*n* = 5) subgroups. As shown in Table 2, no significant differences were observed between the two subgroups in demographic characteristics or etiologies (*p* > 0.05 for each), indicating that the two subgroups were comparable at baseline. Detailed seizure characteristics and EEG findings for each subgroup are also presented in Supplementary Table 2.

**Table 2:** Clinical characteristics of the ACTH-effective subgroup and ACTH-ineffective subgroup of the IESS group.

Variables	ACTH-effective subgroup (*n* = 13)	ACTH-ineffective subgroup (*n* = 5)	*p*-value
Demographics			
Birth weight (g), mean ± SD	3.02 ± 0.64	2.93 ± 1.01	0.870
Age (months), mean ± SD	10.46 ± 4.63	11.40 ± 6.66	0.780
Gender, *n* (%)			
Males	7 (53.8)	4 (80.0)	0.630
Females	6 (46.2)	1 (20.0)	0.630
Gestational age, *n* (%)			
Premature labor	2 (15.4)	2 (40.0)	0.620
Full-term gestation	11 (84.6)	3 (60.0)	0.620
Mode of delivery, *n* (%)			
Natural delivery	2 (15.4)	1 (20.0)	1.000
Cesarean section	11 (84.6)	4 (80.0)	1.000
Feeding situation, *n* (%)			
Breastfeeding	4 (30.8)	2 (40.0)	1.000
Artificial feeding	6 (46.2)	1 (20.0)	0.362
Mixed	3 (23.1)	2 (40.0)	0.580
History of intrauterine distress asphyxia, *n* (%)			
Yes	3 (23.1)	1 (20.0)	1.000
No	10 (76.9)	4 (80.0)	1.000
Etiology			
Structural abnormality on MRI, *n* (%)	3 (23.1)	1 (20.0)	1.000
Genetic etiology, *n* (%)	1 (7.7)	1 (20.0)	0.486

Data are presented as mean ± SD or *n* (%). ACTH, adrenocorticotropic hormone; IESS, infantile epileptic spasms syndrome; MRI, magnetic resonance imaging; SD, standard deviation. *p*-values were calculated using Fisher’s exact test for categorical variables and Mann–Whitney *U* test for continuous variables. Due to the small sample size in the ACTH-ineffective subgroup (*n* = 5), percentages should be interpreted with caution. Detailed seizure characteristics and EEG findings are presented in Supplementary Table 2.

### 3.2 Sequencing quality and shared species analysis

**Figure 1:**
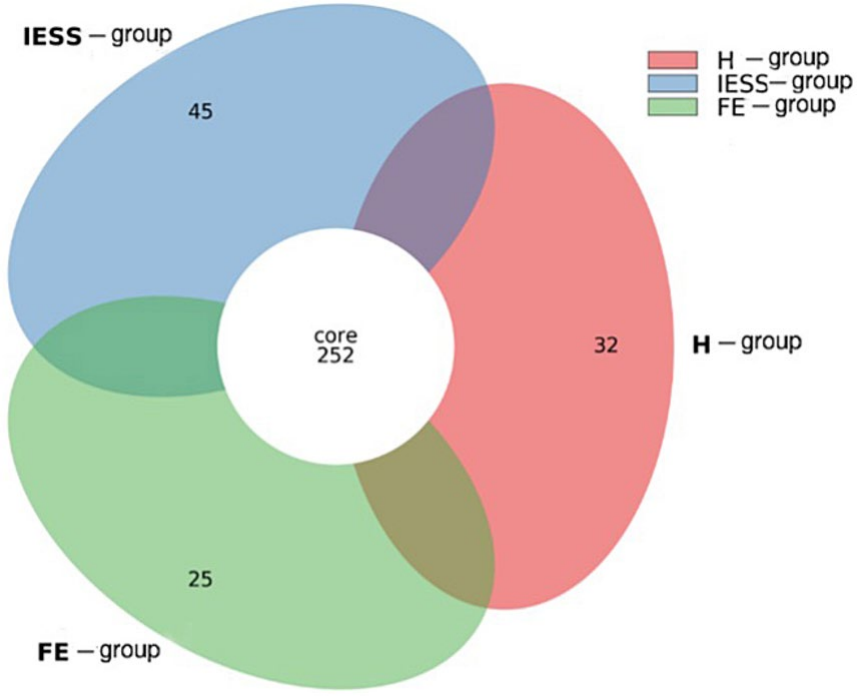
Venn diagram of common or specific gut microbiota species between the IESS and control groups.

16S rDNA gene sequencing was performed on 69 fecal samples. The optimized sequences were clustered into operational taxonomic units (OTUs) based on 97% sequence similarity, yielding 427 OTUs. The representative sequences of each OTU were compared with the reference database; species with the highest similarity and a confidence level of 80% or more were selected, and taxonomic annotations were assigned at the phylum, class, order, family, genus, and species levels. A Venn diagram was constructed to analyze shared species (Figure 1), revealing 45 unique OTUs in the IESS group, 25 in the FE group, 32 in the H group, and 252 OTUs shared among the three groups.

### 3.3 Analysis of α-diversity of gut microbiota

The Chao1, ACE, and observed species indices were used to characterize richness; the Shannon and Simpson indices were used to characterize diversity; and Good’s coverage index was used to characterize sequencing depth (Table 3). No significant differences were observed in the richness indices among the three groups (*p* > 0.05). The Shannon and Simpson indices differed significantly between the IESS and FE groups (*p* < 0.05), while no significant differences were detected between the IESS group and the healthy control group (H group) (*p* > 0.05).

**Table 3:** Comparison of gut microbiota diversity indices between the IESS and control groups [M (P25, P75)].

Groups	Chao1	ACE	Shannon	Simpson	Good’s coverage	Observed species
IESS (*n* = 18)	125.52 (95.41, 158.64)	124.35 (105.40, 145.10)	3.78 (3.44, 3.97)	0.87 (0.84, 0.89)	0.9984 (0.9980, 0.9987)	95.50 (73.50, 108.25)
FE (*n* = 18)	137.46 (105.84, 166.81)	142.24 (118.17, 161.16)	4.18 (3.73, 4.56)	0.90 (0.87, 0.93)	0.9984 (0.9979, 0.9987)	107.5 (87.0, 122.5)
H (*n* = 18)	127.50 (107.89, 149.47)	125.22 (107.87, 136.25)	3.94 (3.67, 4.46)	0.88 (0.85, 0.90)	0.9985 (0.9982, 0.9988)	101 (89, 108)
*p*-value						
IESS vs. H	0.89	0.99	0.14	0.46	0.51	0.41
IESS vs. FE	0.44	0.35	0.01	0.01	0.92	0.19
FE vs. H	0.37	0.40	0.29	0.05	0.46	0.52

^*^Indicates *p* < 0.05.

### 3.4 β-diversity analysis of gut microbiota

**Figure 2:**
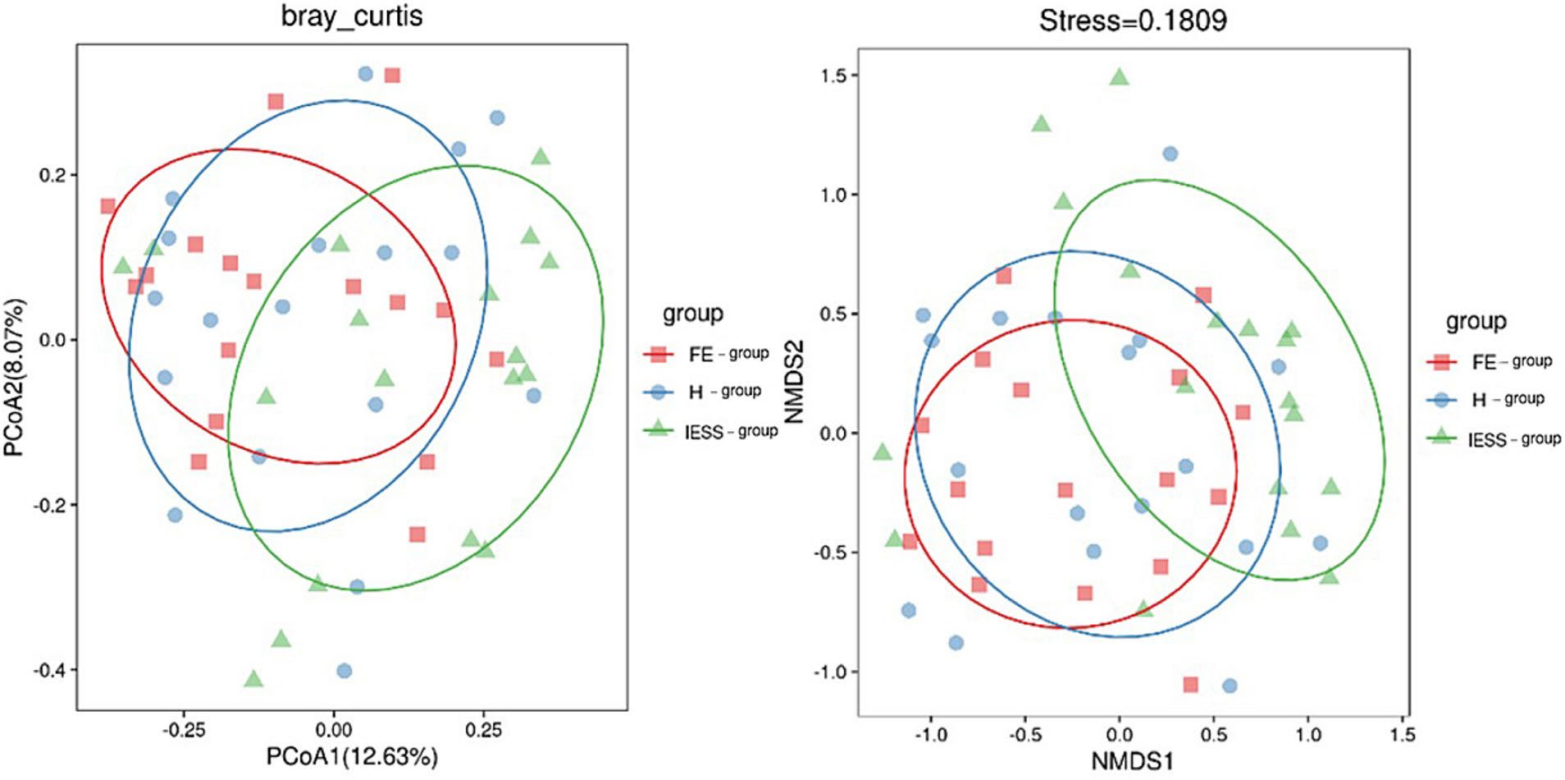
PCoA and NMDS plots of gut microbiota in the IESS and control groups. The dots represent samples; red represents the FE group, blue represents the H group, and green represents the IESS group. The distance between the dots represents the degree of difference between the sample flora, and the farther the distance, the greater the difference between the samples. The PCoA plot shows that the degree of explanation of variation among the three groups by the first principal component is 12.63%, and that by the second principal component is 8.07%. In the NMDS plot, the stress value of the icon is less than 0.2, indicating that NMDS can accurately reflect the degree of difference between samples.

Principal coordinate analysis (PCoA) and non-metric multidimensional scaling (NMDS) analysis were performed based on the Bray–Curtis distance algorithm (Figure 2). The Adonis test indicated a significant difference in gut microbiota composition among the IESS, FE, and H groups (*p* < 0.05).

### 3.5 LDA effect size (LEfSe) analysis of gut microbiota

**Figure 3:**
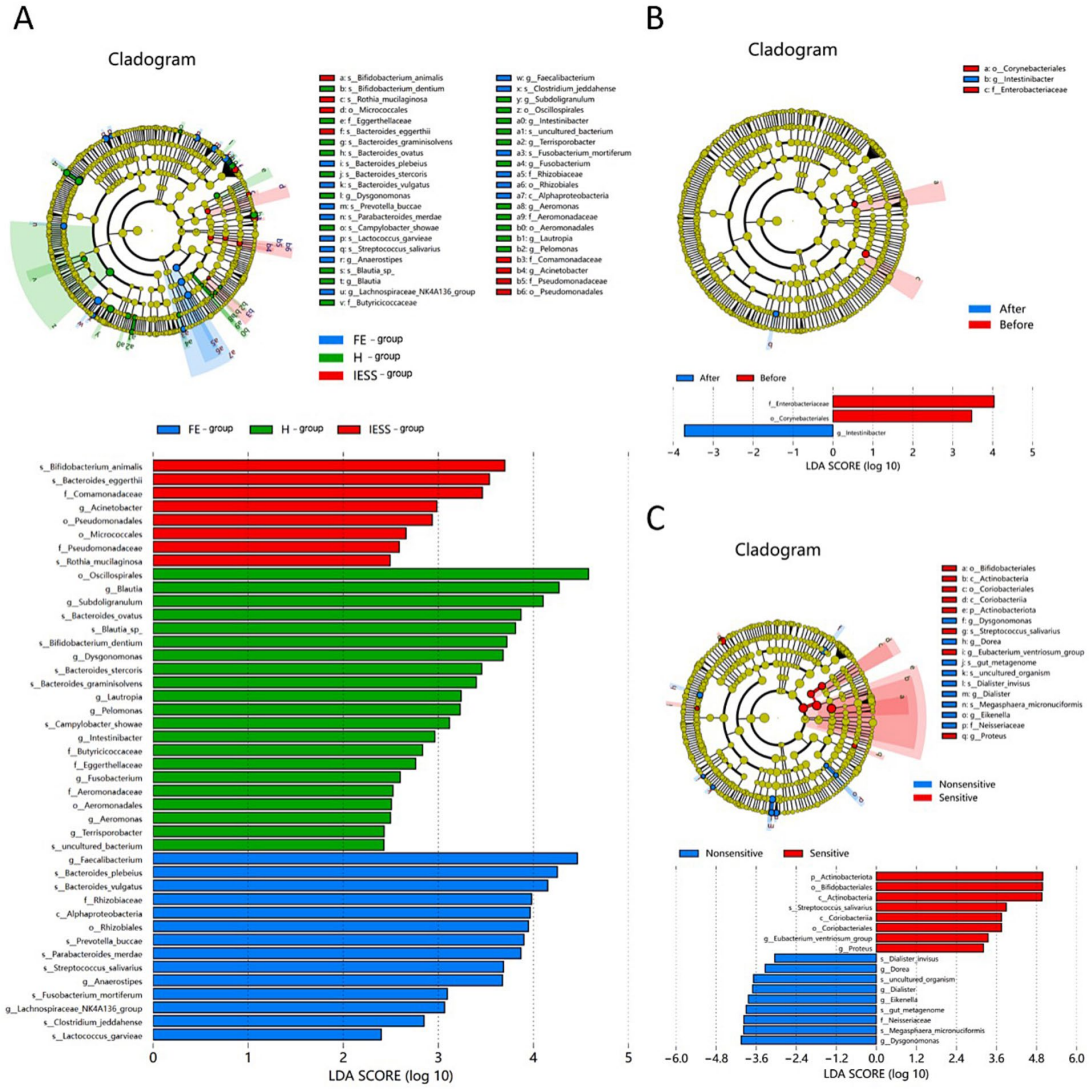
LEfSe evolutionary branching plots and LDA bars of gut microbiota. **(A)** IESS, FE, and H groups. **(B)** Before and after ACTH treatment in the IESS group. **(C)** ACTH-effective subgroup and ACTH-ineffective subgroup of the IESS group. The results of significantly different species are shown for each group. If there were no eligible species in a group, the group is not shown or labeled. In the LDA bar graph, the vertical coordinates represent species with significant differences between groups, and the horizontal coordinates represent a bar graph visualizing the logarithmic score of the LDA analysis for each species. The longer the bar length, the more significant the difference between the species in the group.

LEfSe evolutionary branching plots and LDA bar graphs were plotted (Figure 3), with |LDA| >2 and *p* < 0.05, as the difference screening threshold. *Bifidobacterium animalis*, *Bacteroides eggerthii*, Comamonadaceae, *Acinetobacter*, and Pseudomonadales were significantly enriched in the IESS group, while *Faecalibacterium*, *Bacteroides plebeius*, *Bacteroides vulgatus*, Rhizobiaceae, and Alphaproteobacteria were significantly enriched in the FE group. Moreover, Oscillospirales, *Subdoligranulum*, *Bacteroides ovatus*, and *Blautia* were significantly enriched in the H group. The largest differences in relative abundance were observed for *Bifidobacterium animalis*, *Faecalibacterium*, and Oscillospirales in the IESS, FE, and H groups, respectively.

Before ACTH treatment, Actinobacteria, Bifidobacteriales, *Streptococcus salivarius*, and Coriobacteriia were significantly enriched in the ACTH-effective subgroup, while *Dysgonomonas*, *Megasphaera micronuciformis*, Neisseriaceae, and *Eikenella* were significantly enriched in the ACTH-ineffective subgroup. The greatest differences in relative abundances were observed for Actinobacteriota and *Dysgonomonas* in the ACTH-effective and ACTH-ineffective subgroups, respectively. Moreover, Corynebacteriales and Enterobacteriaceae were significantly enriched before ACTH treatment, while *Intestinibacter* was significantly enriched after ACTH treatment.

### 3.6 Species composition at all levels

**Figure 4:**
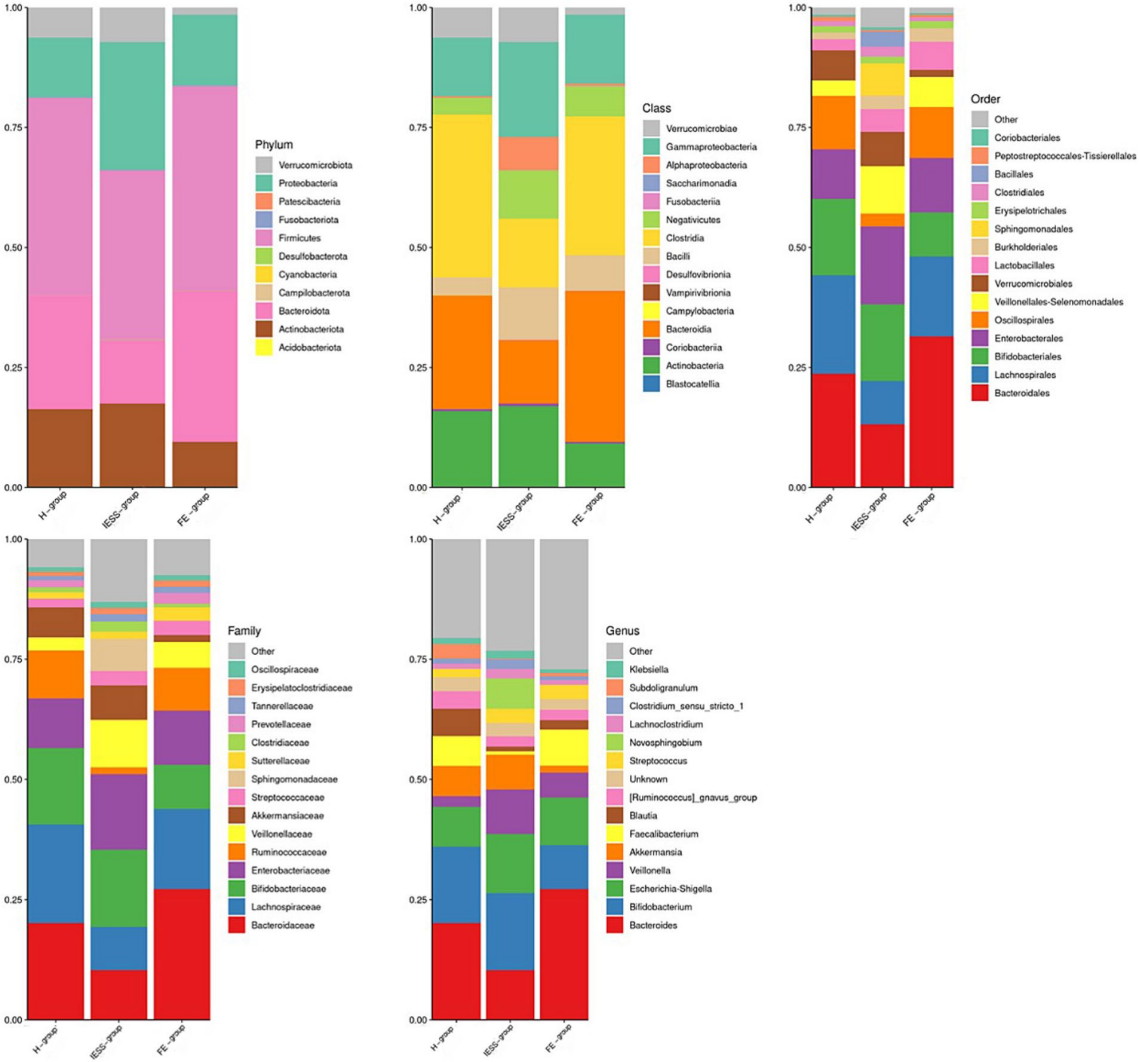
Histograms of gut microbiota species composition between the IESS and control groups.

Metastatic analysis revealed significant differences in gut microbiota composition among the IESS, FE, and H groups at multiple taxonomic levels. Detailed comparisons between IESS and H groups, IESS and FE groups, as well as between ACTH response subgroups, are presented in Figure 4 and Tables 4–6.

**Table 4:** Summary of significantly different bacterial populations of gut microbiota in the IESS and H groups.

Gut microbiota	IESS group (*n* = 18)	H group (*n* = 18)	*p*-value	Hedges’ *g* (95% CI)	Direction
Phylum					
Proteobacteria	0.213 ± 0.040	0.122 ± 0.022	0.049^*^	2.76 [1.85, 3.65]	↑
Class					
Clostridia	0.193 ± 0.032	0.345 ± 0.041	0.009^**^	−4.09 [−5.32, −2.83]	↓
Bacilli	0.085 ± 0.016	0.039 ± 0.011	0.016^*^	3.21 [2.22, 4.18]	↑
Negativicutes	0.107 ± 0.028	0.038 ± 0.010	0.029^*^	3.22 [2.23, 4.19]	↑
Order					
Oscillospirales	0.040 ± 0.011	0.112 ± 0.024	0.010^*^	−3.67 [−4.81, −2.51]	↓
Lachnospirales	0.120 ± 0.022	0.211 ± 0.029	0.024^*^	−3.41 [−4.47, −2.33]	↓
Veillonellales-Selenomonadales	0.103 ± 0.028	0.035 ± 0.011	0.038^*^	3.12 [2.12, 4.10]	↑
Family					
Lachnospiraceae	0.120 ± 0.022	0.211 ± 0.029	0.015^*^	−3.45 [−4.52, −2.37]	↓
Ruminococcaceae	0.022 ± 0.008	0.101 ± 0.022	0.003^**^	−4.59 [−5.91, −3.24]	↓
Veillonellaceae	0.104 ± 0.028	0.029 ± 0.009	0.017^*^	3.53 [2.45, 4.59]	↑
Genus					
*Faecalibacterium*	0.011 ± 0.007	0.065 ± 0.018	0.008^**^	−3.84 [−4.99, −2.66]	↓
*Blautia*	0.014 ± 0.006	0.060 ± 0.021	0.013^*^	−2.90 [−3.86, −1.92]	↓
*Veillonella*	0.097 ± 0.029	0.023 ± 0.010	0.021^*^	3.36 [2.33, 4.36]	↑

Data are presented as mean ± standard deviation. Effect sizes (Hedges’ *g*) and their 95% confidence intervals (CI) were calculated based on the non-central *t*-distribution. All *p*-values were derived from two-tailed independent samples *t*-tests.

Statistical significance is indicated as follows: ^*^p < 0.05 and ^**^p < 0.01.

Hedges’ *g* interpretation (Cohen, 1988): |*g*| ≥ 0.8 indicates a large effect; |*g*| ≥ 1.20 indicates a very large effect.

Arrows indicate the direction of change in the IESS group relative to the healthy control group: ↑, significantly higher; ↓, significantly lower.

**Table 5:** Summary of significantly different bacterial populations of the gut microbiota in the IESS and FE groups.

Gut microbiota	IESS group (*n* = 18)	FE group (*n* = 18)	*p*-value	Hedges’ *g* (95% CI)	Direction
Phylum					
Actinobacteriota	0.195 ± 0.045	0.086 ± 0.019	0.020^*^	3.06 [2.08, 4.03]	↑
Class					
Actinobacteria	0.186 ± 0.041	0.083 ± 0.019	0.016^*^	3.13 [2.14, 4.11]	↑
Clostridia	0.193 ± 0.032	0.299 ± 0.037	0.047^*^	−3.00 [−3.97, −2.02]	↓
Order					
Oscillospirales	0.040 ± 0.011	0.108 ± 0.022	0.004^**^	−3.76 [−4.90, −2.59]	↓
Bifidobacteriales	0.183 ± 0.042	0.083 ± 0.019	0.034^*^	2.99 [2.01, 3.96]	↑
Family					
Ruminococcaceae	0.022 ± 0.008	0.089 ± 0.021	0.004^**^	−4.06 [−5.28, −2.81]	↓
Bifidobacteriaceae	0.183 ± 0.042	0.083 ± 0.019	0.034^*^	2.99 [2.01, 3.96]	↑
Genus					
Bifidobacterium	0.183 ± 0.042	0.083 ± 0.019	0.033^*^	2.99 [2.01, 3.96]	↑

Data are shown as mean ± SD. *p*-values were derived from two-tailed independent samples *t*-tests. Effect sizes (Hedges’ *g*) and their 95% CIs were calculated as described in Table 4.

^*^p < 0.05 and ^**^p < 0.01.

Hedges’ *g* interpretation: |*g*| ≥ 0.8 indicates a large effect; |*g*| ≥ 1.20 indicates a very large effect.

Arrows indicate the direction of change in the IESS group relative to the FE group: ↑, significantly higher; ↓, significantly lower.

**Table 6:** Summary of significantly different bacterial populations of the gut microbiota in the ACTH-effective and ACTH-ineffective groups before treatment.

Gut microbiota	ACTH-effective group (*n* = 13)	ACTH-ineffective group (*n* = 5)	*p*-value	Hedges’ *g* (95% CI)	Direction
Phylum					
Actinobacteriota	0.252 ± 0.055	0.046 ± 0.018	0.040^*^	4.39 [2.30, 6.45]	↑
Class					
Actinobacteria	0.240 ± 0.049	0.046 ± 0.018	0.040^*^	4.47 [2.35, 6.55]	↑
Order					
Bifidobacteriales	0.239 ± 0.049	0.038 ± 0.016	0.039^*^	4.65 [2.46, 6.80]	↑
Lactobacillales	0.066 ± 0.018	0.019 ± 0.014	0.022^*^	2.71 [1.26, 4.13]	↑
Family					
Bifidobacteriaceae	0.239 ± 0.049	0.038 ± 0.016	0.017^*^	4.65 [2.46, 6.80]	↑
Genus					
Bifidobacterium	0.239 ± 0.049	0.037 ± 0.016	0.009^**^	4.73 [2.51, 6.92]	↑

Data are shown as mean ± SD. *p*-values were derived from two-tailed independent samples *t*-tests. Effect sizes (Hedges’ *g*) and their 95% CIs were calculated as described in Table 4.

^*^p < 0.05 and ^**^p < 0.01.

Hedges’ *g* interpretation: |*g*| ≥ 0.8 indicates a large effect; |*g*| ≥ 1.20 indicates a very large effect.

Arrows indicate the direction of change in the ACTH-effective group relative to the ACTH-ineffective group: ↑, significantly higher; ↓, significantly lower.

The relative abundances of Proteobacteria and Actinobacteriota in the IESS group were higher (*p* < 0.05), but that of Ruminococcaceae was lower (*p* < 0.01) than those in the FE and H groups, with very large effect sizes in most comparisons [e.g., Proteobacteria vs. H: Hedges’ *g* = 2.76 (1.85, 3.65); Ruminococcaceae vs. H: g = −4.59 (−5.91, −3.24)]. Further, the IESS group had a higher abundance of Veillonellaceae (*p* < 0.05) and lower abundance of Lachnospiraceae (*p* < 0.05) and *Faecalibacterium* (*p* < 0.01) compared with those in the H group, all with very large effects [Veillonellaceae: *g* = 3.53 (2.45, 4.59); Lachnospiraceae: *g* = −3.45 (−4.52, −2.37); *Faecalibacterium*: *g* = −3.84 (−4.99, −2.66)].

Before ACTH treatment, the abundances of Actinobacteria (phylum and order levels) and Bifidobacteriales (order, family, and genus levels) were lower in the ACTH-ineffective subgroup than in the ACTH-effective subgroup (*p* < 0.05), all with very large effect sizes [Hedges’ *g* ranging from 4.39 to 4.73; 95% CIs (2.30–6.92)], as detailed in Table 6.

ACTH treatment resulted in decreased abundance of Bacilli at the order, family, and genus levels in the IESS group (*p* < 0.05). These taxa, present at low levels before treatment (0.003–0.007), became completely undetectable in all 15 infants after treatment, with very large effect sizes [Hedges’ *g* = 1.38, 95% CI (0.68, 2.07)].

### 3.7 Functional prediction of gut microbiota

**Figure 5:**
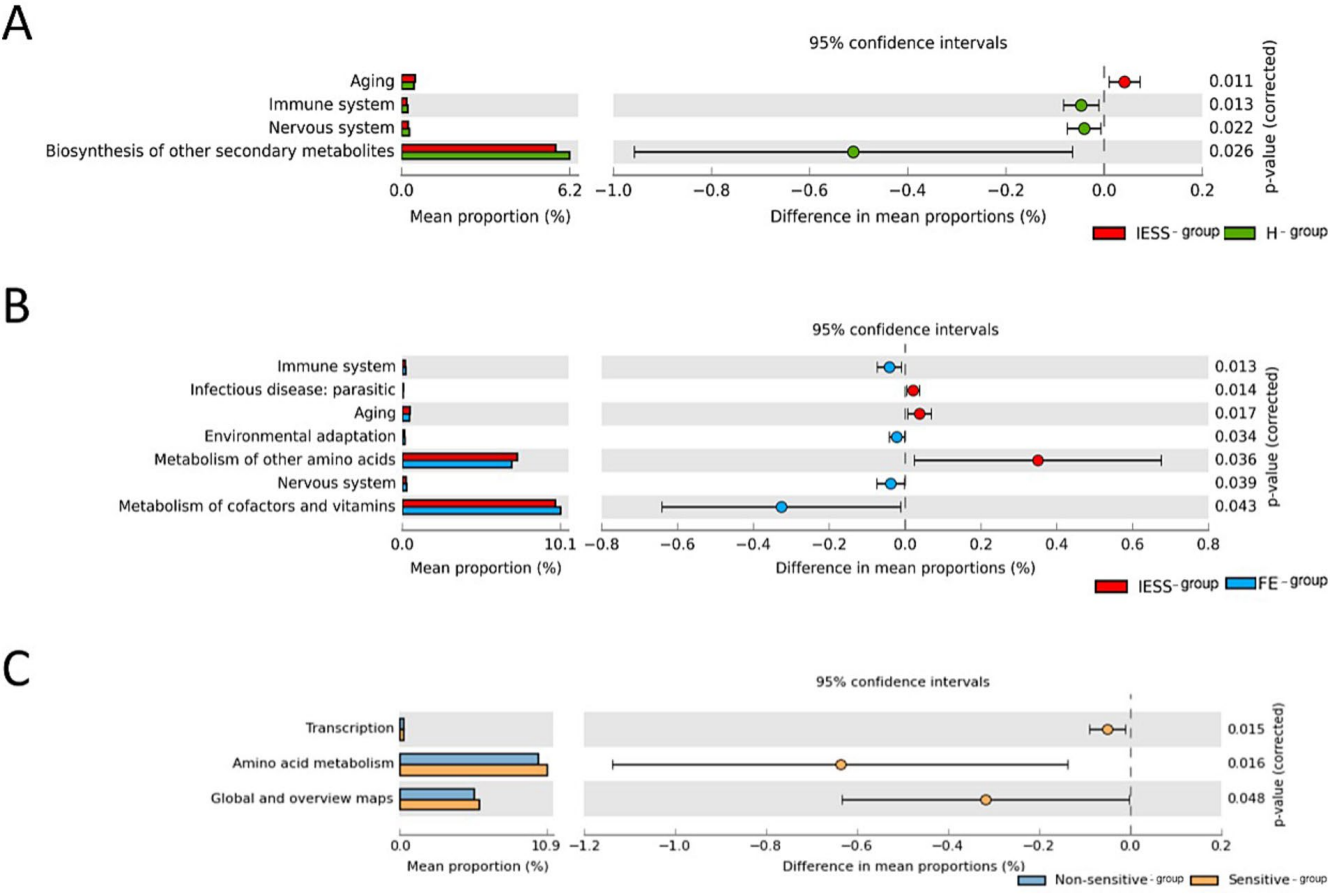
Comparison of functional differences in the gut microbiota at the KEGG-L2 level. **(A)** IESS group versus FE group. **(B)** IESS group versus H group. **(C)** ACTH-effective subgroup versus ACTH-ineffective subgroup of the IESS group.

**Figure 6:**
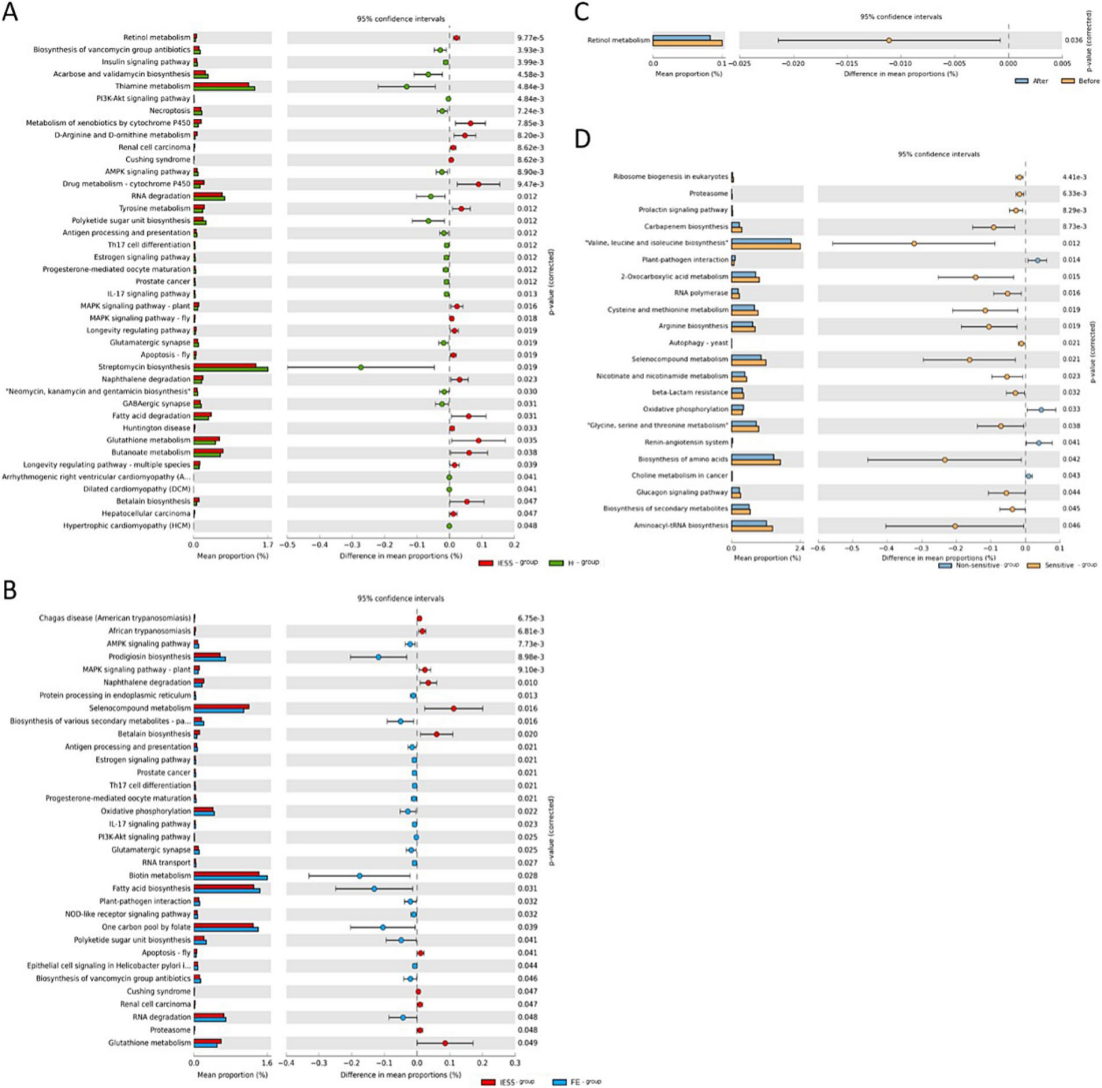
Comparison of functional differences in the gut microbiota at the KEGG-L3 level. **(A)** IESS group versus H group. **(B)** IESS group versus FE group. **(C)** Before and after ACTH treatment in the IESS group. **(D)** ACTH-effective subgroup versus the ACTH-ineffective subgroup of the IESS group.

We performed an in-depth analysis of the metabolic functions of the gut microbiota in the IESS, FE, and H groups using the PICRUSt2 software. We then used the Kyoto Encyclopedia of Genes and Genomes (KEGG) database to assess metabolic pathways and determine metabolic pathway differences at three different levels (level 1 to level 3). The results of functional annotation based on KEGG database using STAMP for differential analysis to predict the function of different species between two groups are shown (Figures 5, 6). There were differences in the functions of multiple metabolic pathways at the KEGG-L2 and KEGG-L3 levels (*p* < 0.05). However, it should be noted that these functional differences represent predictive inferences based on 16S rDNA sequencing data. Specifically, prior to ACTH treatment, transcriptional regulation and amino acid metabolism pathways decreased in the ACTH-ineffective subgroup compared with those in the ACTH-effective subgroup (*p* < 0.05). After ACTH treatment, the function of the retinol metabolism pathway decreased in the IESS group (*p* < 0.05).

### 3.8 Coexistence network analysis of gut microbiota

**Figure 7:**
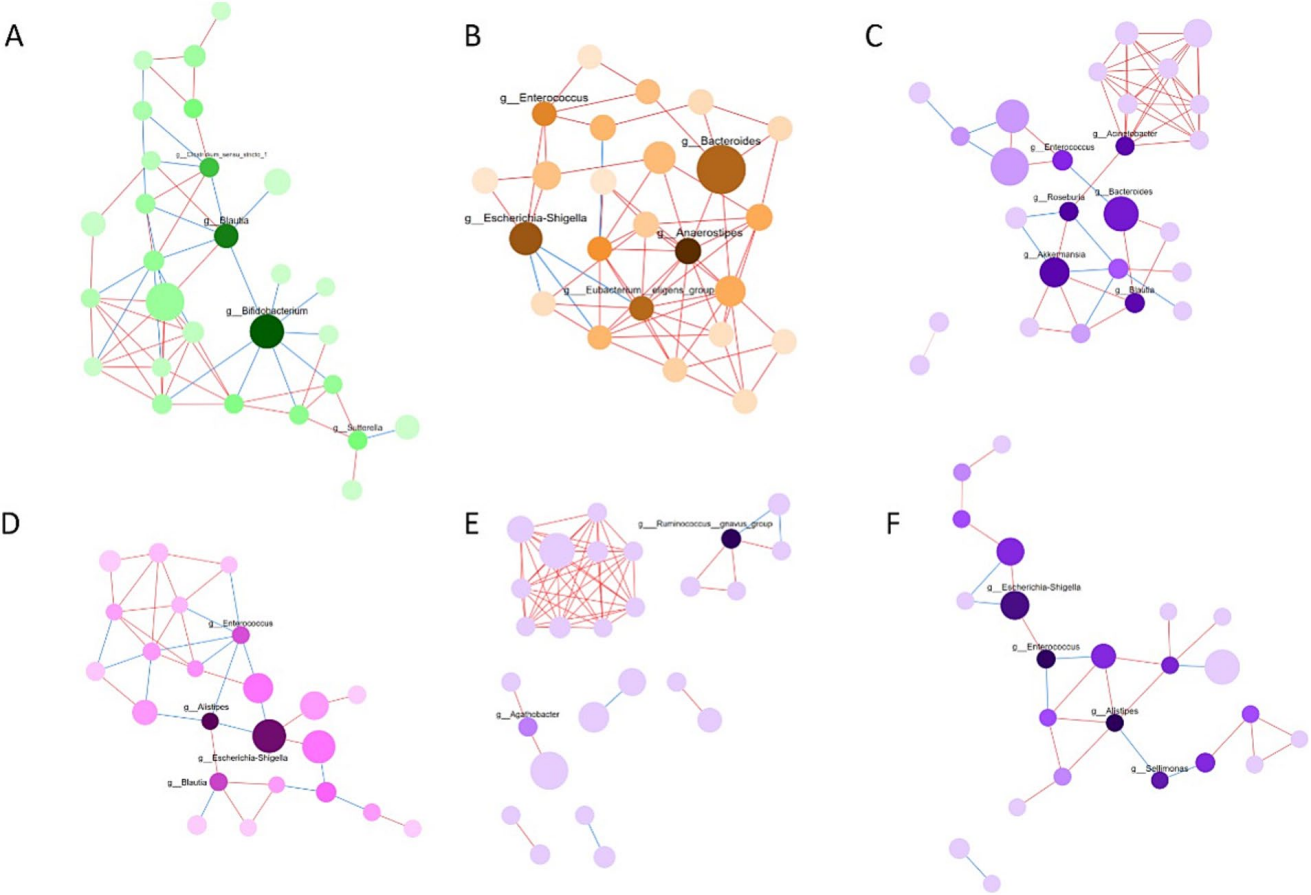
Network analysis of gut microbiota coexistence. **(A)** H group. **(B)** FE group. **(C)** IESS group. **(D)** IESS group after ACTH treatment. **(E)** ACTH-ineffective subgroup of the IESS group. **(F)** ACTH-effective subgroup of the IESS group. Each node represents a genus, and the size of the node represents the relative abundance of the genus; the darker the color of the node, the greater the mediating centrality of the genus, indicating that it has more influence on the ecology and is able to connect to other nodes more effectively. The lines between the circles indicate that the correlation between these two species is significant (*p* < 0.05), the red line represents a positive correlation, and the blue color represents a negative correlation.

We constructed co-occurrence networks at the genus level in the IESS, FE, and H groups (Figure 7). Structural aggregation of the gut microbiota network was weaker in the IESS than in the FE or H group. Before ACTH treatment, the ACTH-ineffective subgroup showed weaker aggregation of the flora network than the ACTH-effective subgroup. After ACTH treatment, the aggregation of gut microbiota network structure was enhanced in the IESS group.

## 4 Discussion

This study found that although there were no significant differences in gut microbial richness and coverage among the IESS, FE, and H groups, α-diversity was significantly lower in the IESS group than in the FE group, while no such significant difference was observed between the IESS and H groups. This pattern suggests that the observed α-diversity differences may reflect heterogeneity between epilepsy subtypes rather than a specific dysbiosis in IESS patients relative to that in healthy individuals. Furthermore, statistically significant differences in β-diversity were observed among the three groups. This finding differs from that of Lee et al. (22), who reported higher diversity in healthy controls than in epilepsy patients; such discrepancies may be influenced by factors including study population, epilepsy subtype, sample size, and geographic location. Besides, although age differences between groups were not statistically significant, the mean age in the IESS group was lower than that in the FE group. Given that the gut microbiota of infants aged 0–24 months undergoes rapid physiological maturation, even minor age differences may influence microbial composition and diversity. Conversely, the Venn diagram revealed substantial sharing of OTUs across the three groups. This supports the interpretation that observed intergroup differences likely represent relative abundance shifts within a broadly overlapping core microbial community.

Our results revealed that the relative abundances of Proteobacteria and Actinobacteriota in the IESS group were higher than those in FE and H groups. The specific enrichment of this phylum in epilepsy patients was further supported by Arulsamy et al. (23). Proteobacteria is a diverse group of bacteria and its abnormal proliferation is closely related to intestinal microecological imbalance and formation of an inflammatory microenvironment (24). For Actinobacteriota, our study presents a noteworthy contradiction. In general, Actinobacteriota is considered a beneficial bacteria. However, we found a trend of elevated Actinobacteria abundance in IESS patients, which differed from the protective hypothesis proposed by Şafak et al. (25).

Ruminococcaceae abundance in the IESS group was significantly lower than that in the FE and H groups. Ruminococcaceae levels may influence neonatal neurodevelopment by negatively regulating N-acetylaspartate levels in the brain and affecting serum cortisol levels. It is also associated with the development of neurodevelopmental disorders (26). Additionally, as serotonin inhibits T-type calcium channels and reduces electrical burst activity, an increase in Ruminococcaceae may facilitate epileptiform discharges due to serotonin reduction (27). However, in our IESS cohort, Ruminococcaceae abundance decreased, suggesting that this may be a unique gut microbiota change, or there are unknown regulatory mechanisms underlying IESS.

Herein, the IESS group had a higher Veillonellaceae abundance and lower Lachnospiraceae than the H group. Veillonellaceae belongs to the phylum Firmicutes, which enhances neuronal signaling by increasing neurotransmitter levels. This augmentation may lead to abnormal brain function and neuron over-discharge, which, in turn, may trigger seizures. Moreover, Veillonellaceae is a potential risk factor for childhood-absence epilepsy (28). It was also significantly associated with an increase in fearful behavior during a non-social fear paradigm (29). Further, Veillonellaceae abundance may correlate with the level of activity in specific brain regions, which may be associated with cognitive decline. However, the participants in previous studies were mainly adults, limiting comparability to our infantile population (30). Cognitive decline is pronounced in IESS patients compared with that in a healthy group of the same age. Here, we also observed a significant decrease in the relative abundance of Lachnospiraceae in the IESS group compared with that in the H group, which differs from the previous reports. As a core group of anaerobic bacteria, Lachnospiraceae is involved in host metabolic regulation starting from infancy, and its functions exhibit significant heterogeneity. On the one hand, Lachnospiraceae can maintain intestinal homeostasis through the production of short-chain fatty acids by fermenting dietary fibers. On the other hand, its aberrant colonization is associated with a variety of inflammation-related diseases, such as metabolic syndrome and neuropsychiatric disorders (31). Notably, You et al. (9) found an elevated abundance of Lachnospiraceae in 14 cases of IESS and a positive correlation with the level of corticotropin-releasing hormone, which may promote epileptic seizures, suggesting that Lachnospiraceae is involved in seizures through HPA axis regulation.

*Faecalibacterium* abundance was also significantly lower in the IESS group than in the H group. *Faecalibacterium* plays an important role in the immune system, intestinal barrier, and microbiota regulation. Butyrate, a major metabolite of this genus, has multiple effects on host physiology (32). *Faecalibacterium* has a variety of beneficial effects, promising to be a next-generation probiotic or active biotherapeutic product, and highlighting its importance in gut microbiota health and disease prevention (33). *Faecalibacterium* abundance is usually reduced in neurological disorders, such as attention deficit hyperactivity disorder and Parkinson’s disease, but is higher in children with autism spectrum disorder (34). Moreover, *Faecalibacterium* abundance was higher in epilepsy patients than in healthy controls, although this was not significantly associated with disease severity (35). However, current findings on the association between *Faecalibacterium* and epilepsy are controversial, which may be attributed to a variety of factors, such as age and region, necessitating further research.

Functional prediction analysis based on PICRUST2 revealed no significant differences between the IESS group and two control groups at the KEGG pathway L1 level. However, at the L2 level, a significant advantage in aging function and a lower performance in immune and neurological functions were observed in the IESS group than in the FE and H groups. Furthermore, the ACTH-ineffective subgroup exhibited lower levels of transcription, amino acid metabolism, and global function. At level 3, significant differences in multiple metabolic pathways were observed between the IESS and two control groups. The IESS group showed higher enrichment in retinol metabolism, naphthalene degradation, betaine biosynthesis, and glutathione metabolism than the FE and H groups, with retinol metabolism differing only between the IESS and H groups, suggesting that the retinol metabolism pathway may play a role during IESS development.

Assessment of gut microbial network revealed significant structural differences among the IESS, FE, and H groups, as evidenced by weakened network aggregation, reduced genus connectivity, and a lack of dominant genera.

It was observed that the abundances of Actinobacteria (phylum and order levels), Bifidobacteria (order, family, and genus levels), and Lactobacillales (order level) were significantly lower in the ACTH-ineffective than in the ACTH-effective subgroups before ACTH treatment. Bifidobacteria, belonging to Actinobacteriota, is a beneficial bacteria in the human intestinal tract, especially during infancy and early childhood. We found that, before ACTH treatment, Bifidobacteria abundance in the ACTH-effective subgroup was significantly higher than that in the ACTH-ineffective subgroup, suggesting that the absence of Bifidobacteria may play an important role in the poor efficacy in the ACTH-ineffective subgroup. Elevated Bifidobacteria abundance is more likely to benefit children with drug-resistant epilepsy through ketogenic diet therapy (36), which is consistent with the results of this study. Additionally, Lactobacillales inhibits the growth and reproduction of harmful bacteria to maintain the stability of intestinal microecology. In the IESS model, seizures were successfully attenuated by regulating the gut microbiota, and Lactobacillales increase, paralleled with alleviation of spasticity symptoms, was observed after antiepileptic therapy (37).

After ACTH treatment, microbial network aggregation and genus connectivity significantly improved in the IESS group, suggesting that ACTH treatment may play a role in remodeling the structure of the intestinal microbial network. Additionally, before ACTH treatment, the ACTH-effective subgroup had microbial networks with stronger aggregation and higher genus connectivity and was more likely to form dominant genera. The ACTH-ineffective subgroup showed the opposite network characteristics, strongly suggesting that the health status of microbial network is closely related to the efficacy of ACTH treatment. The gut microbiota of healthy infants reportedly possesses higher complexity and tighter connectivity than that of epilepsy children (38), which is consistent with our results. Taken together, we hypothesize that the microbial network characteristics of IESS are significantly associated with disease severity and response to ACTH therapy, which may provide new perspectives for understanding the mechanisms underlying IESS and lead to the development of novel therapeutic strategies based on gut microbiota modulation.

Bacilli belong to the phylum Firmicutes, a common member of the intestinal tract that maintains intestinal microecological balance and is potentially pathogenic. Herein, their abundance decreased after ACTH treatment at the order, family, and genus levels, suggesting that the abundance of bacilli may be associated with IESS improvement. *Bacillus subtilis* reportedly plays a potential role in alleviating resistance to antiepileptic drugs in patients with drug-resistant epilepsy via modulation of ABCB1 transporter proteins (39). ACTH treatment may modulate flora composition in IESS patients. However, changes in flora composition vary across different studies.

Additionally, retinol metabolism was significantly reduced in the IESS group after ACTH treatment. Unesterified docosahexaenoic acid reportedly regulates synapse formation and excitatory synaptic transmission *in vivo* through retinoid X receptor alpha (RXRα) signaling, suggesting that endogenous RXRα plays an important role in synaptogenesis in cortical pyramidal neurons (40). However, these findings should be interpreted with caution. The functional differences reported herein, particularly those related to transcriptional regulation, amino acid metabolism, and retinol metabolism, are derived from predictive inferences based on PICRUSt2. Given the small sample size of the ACTH subgroup and the inherently multi-step inferential nature of 16S-based functional prediction, these results should be regarded as hypothesis-generating signals and require validation in future studies using larger cohorts, supplemented by techniques such as metagenomics or metabolomics.

Herein, the predominant gut microbiota before ACTH treatment was Corynebacteriales and Enterobacteriaceae, which changed to *Intestinibacter* after ACTH treatment. *Corynebacterium diphtheriae* (Corynebacteriales) may affect neurological function by releasing neurotoxins or activating the entero-encephalic axis (41). Members of the family Enterobacteriaceae, including *Escherichia coli*, *Salmonella*, and *Shigella*, induce inflammation and increase intestinal permeability (42). Enterobacteriaceae is helpful for maintaining intestinal microecological balance, inhibiting the growth and reproduction of harmful bacteria, and participating in the metabolic absorption of nutrients. It has been hypothesized that *Intestinibacter* is indirectly involved in mucus degradation and resistance to colonization by enterotoxin-producing bacteria (43). Furthermore, Enterobacteriaceae abundance increases in epilepsy in adults (35).

### 4.1 Limitations

This study has several limitations. First, the modest sample size may limit statistical power and generalizability; larger independent cohorts are needed to validate our findings. Second, although LEfSe with LDA thresholds was used to identify robust differential features, the absence of multiple testing correction for all pairwise comparisons may increase the risk of false-positive findings. However, the consistency of observed differences across multiple taxonomic levels suggests biological relevance. Third, 16S rRNA sequencing provides limited taxonomic resolution at the species level, and functional predictions from PICRUSt2 are inferential rather than direct measurements. Accordingly, species-level findings should be interpreted with caution and confirmed by metagenomic sequencing in future studies. Fourth, the cross-sectional design precludes causal inference, and our observations regarding ACTH efficacy prediction should be considered hypothesis-generating, warranting validation in prospective cohorts. Fifth, while the IESS group included patients with diverse etiologies, the majority had unknown etiology, and no significant differences in etiological distribution were observed between the ACTH-effective and ACTH-ineffective subgroups. Finally, this was a single-center study with a referral-based cohort, and systematic long-term follow-up was not feasible for all participants. Future multicenter studies with longitudinal follow-up are needed to determine whether baseline gut microbiota profiles are associated with long-term clinical outcomes.

## 5 Conclusion

Gut microbiota profiles in children with IESS suggested a potential association with response to ACTH therapy. These preliminary findings, while requiring validation in larger independent cohorts, highlight a potential role for the gut microbiota in the pathophysiology of IESS and underscore the need for further multi-omic investigations to elucidate underlying mechanisms.

## Data Availability

The data supporting the findings of this study are available upon request from the corresponding author. The data are not publicly available because of privacy and ethical restrictions.
