# Supplementary material for: Gut microbiota dynamics and its impact on the efficacy of ACTH therapy in infantile epileptic spasms syndrome
Source: Front Neurol. 2026 Apr 15;17:1804171. doi: 10.3389/fneur.2026.1804171 (PMC13124983; doi:10.3389/fneur.2026.1804171)
Supplement: Supplementary file 1 [file Data_Sheet_1.pdf]

Supplementary Table 1. Clinical characteristics of patients with infantile epileptic spasms syndrome (IESS) and focal epilepsy (FE)

Clinical feature	IESS (n=18)	FE (n=18)
**Age at onset (months)**	6.1 ± 4.9	11.9 ± 6.9
**Epileptic spasms**		
Spasm type		
Flexor	17	—
Extensor	0	—
Mixed	1	—
Symmetry		
Symmetric	17	—
Asymmetric	1	—
Cluster frequency		
< 10 clusters/day	13	—
≥ 10 clusters/day	5	—
Cluster duration		
< 5 min	17	—
≥ 5 min	1	—
Total daily spasm count		
< 100	14	—
≥ 100	4	—
**Focal seizures**		
Focal motor seizures		
Focal tonic	3	11
Focal clonic	1	6
Focal epileptic spasms	0	1
Daily frequency of focal seizures		
< 5/day	4	13
≥ 5/day	0	5
Duration of focal seizures		
< 1 min	4	10
1–5 min	0	5
≥ 5 min to ≤ 30 min	0	3
**EEG features**		
Hypsarrhythmia type		
Typical hypsarrhythmia	16	0
Modified hypsarrhythmia	2	0
Ictal discharge origin of focal seizures		
Frontal	1	8
Temporal	2	5
Central-parietal	1	4
Occipital	0	1
**Development**		
Delayed	14	2
Normal	4	16

Note: Data are presented as number of cases or mean ± standard deviation. IESS, infantile epileptic spasms syndrome; FE, focal epilepsy. —, not applicable.

Supplementary Table 2. Clinical characteristics of responders and non-responders with infantile epileptic spasms syndrome (IESS)

Clinical feature	Responders (n=13)	Non-responders (n=5)
**Age at onset (months)**	6.6 ± 5.4	5.0 ± 5.5
**Epileptic spasms**		
Spasm type		
Flexor	12	5
Extensor	0	0
Mixed	1	0
Symmetry		
Symmetric	12	5
Asymmetric	1	0
Cluster frequency		
< 10 clusters/day	10	3
≥ 10 clusters/day	3	2
Cluster duration		
< 5 min	13	4
≥ 5 min	0	1
Total daily spasm count		
< 100	11	3
≥ 100	2	2
**Focal seizures**		
Focal motor seizures		
Focal tonic	3	0
Focal clonic	1	0
Focal epileptic spasms	0	0
Daily frequency of focal seizures		
< 5/day	4	0
≥ 5/day	0	0
Duration of focal seizures		
< 1 min	4	0
1–5 min	0	0
≥ 5 min to ≤ 30 min	0	0
**EEG features**		
Hypsarrhythmia type		
Typical hypsarrhythmia	11	5
Modified hypsarrhythmia	2	0
Ictal discharge origin of focal seizures		
Frontal	1	0
Temporal	2	0
Central-parietal	1	0
Occipital	0	0
**Development**		
Delayed	9	5
Normal	4	0

Note: Data are presented as number of cases or mean ± standard deviation. Responders were defined as patients achieving seizure freedom with EEG improvement, or a reduction in seizure frequency of ≥50% after treatment; non-responders were defined as patients with a reduction in seizure frequency of <50% or no EEG improvement.
